# Surgery for adrenal gland disease. Experience of a tertiary center

**DOI:** 10.1016/j.sopen.2026.01.009

**Published:** 2026-02-10

**Authors:** Gaia Cicioni, Immacolata Iannone, Daniele Crocetti, Mariarita Tarallo, Paolo Sapienza, Giuseppe Cavallaro, Giorgio De Toma, Luigi Petramala, Claudio Letizia, Maria Irene Bellini

**Affiliations:** a Department of Surgery, Sapienza University, Rome, Italy; b Department of Translational and Precision Medicine, Sapienza University, Rome, Italy; c Department of Medical and Cardiovascular Sciences, Sapienza University, Rome, Italy

## Abstract

**Introduction:**

Laparoscopic adrenalectomy and robotic adrenalectomy are increasingly accepted methods for removing adrenal lesions, especially for benign conditions. This study investigated the evolution of surgical techniques and patient characteristics at a tertiary centre during the transition from open to minimally invasive surgery.

**Patients and methods:**

The analysis included all adrenal surgery cases referred to our institution between January 2009 and June 2025. The following were recorded for each patient: demographics, diagnosis, surgical approach, intraoperative blood loss, histology, length of hospital stays, and complications.

**Results:**

A total of 292 adrenalectomies were performed (56% female; mean age 54.5 ± 12.6 years). The left adrenal was more frequently affected (59%), and the mean tumor size was 46.8 ± 25.1 mm. Indications included primary hyperaldosteronism (37%), Cushing's syndrome (28%), pheochromocytoma (15%), adrenal cysts or myelolipomas (17%), adrenocortical carcinomas (4%), and adrenal metastases (2%). Laparoscopy was the most common approach (76%), followed by open (10%) and robotic adrenalectomy (9%); overall 7 (2%) patients required conversion to open surgery and postoperative morbidity was 7%. Minimally invasive procedures were associated with shorter operative times, less blood loss, and shorter in-hospital stays when compared to open surgery (*p* < 0.05). Robotic adrenalectomy had the lowest intraoperative blood loss, while laparoscopy had the fastest operative time.

**Conclusions:**

Minimally invasive adrenalectomy is safe and effective for adrenal tumors. In our series, robotic-assisted surgery is becoming increasingly prevalent and has excellent results; however, its implementation needs to be balanced with the associated costs.

## Graphical abstract

**Figure:**
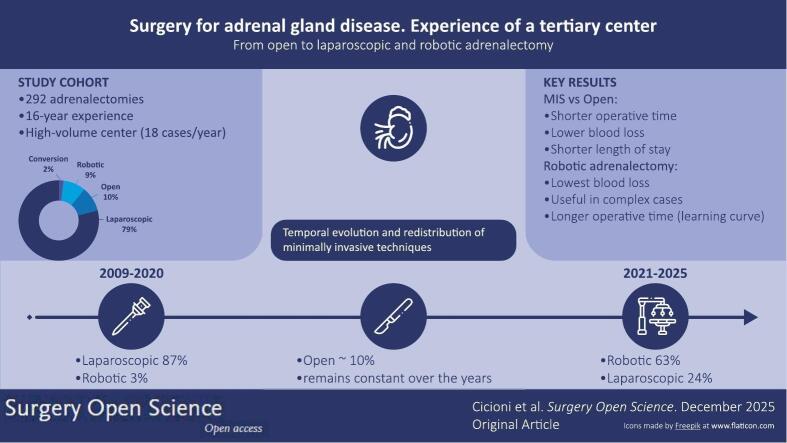

## Introduction

Adrenalectomy is a technically challenging surgical procedure, particularly when performed through a minimally invasive approach [1]. Minimally invasive surgery (MIS), incorporating both laparoscopic and robotic techniques, is now the preferred method for adrenalectomy.

The reported prevalence of adrenal incidentaloma in autopsy series is approximately 2% [2]. Consequently, adrenal surgery is becoming increasingly centralized within high-volume referral centres, a strategy that has been shown to enhance surgical expertise and optimize clinical outcomes. This centralization is especially relevant in challenging cases, where selecting the most suitable surgical approach, i.e. laparoscopic, robotic or open, through a balanced assessment.

Recommendations for identifying a tertiary centre for adrenalectomy suggest a threshold of six procedures per year [3]. However, some recent reports suggest a higher threshold, such as 12 or 20 procedures per year, particularly for malignant diseases, due to increased morbidity and a higher risk of conversion from MIS [4].

Minimally invasive adrenalectomy is typically recommended for adrenal masses measuring up to 7 cm in diameter. However, both laparoscopic and robotic approaches are increasingly being used for larger tumors, particularly in high-volume centres. Laparoscopic adrenalectomy remains technically challenging due to the limited working space, lack of 3D vision, and restricted instrument mobility [5]. These limitations have sparked an ongoing debate about whether open adrenalectomy should remain the standard procedure for patients with suspected adrenocortical carcinoma (ACC), given that conversion from laparoscopy to open surgery appears to reduce overall survival rates [6]. In contrast, robotic surgery offers high-definition 3D visualization, magnification, tremor filtration, and instruments with seven degrees of freedom. These features are associated with lower conversion rates and could extend the use of minimally invasive surgery to more complex cases.

While both minimally invasive approaches appear to be equivalent for benign conditions, the robotic platform may have significant advantages in overcoming the innate limitations of laparoscopy when surgery is planned for malignant adrenal disease [7].

We report our experience over sixteen years, characterized by the transition from open surgery to MIS, and reflect on the fine line between maintaining patient safety and advancing through innovation.

## Patients and methods

All patients who underwent adrenalectomy at our Department between January 2009 and September 2025 were included in the present analysis. The indication for surgery was established by a multidisciplinary team based on clinical evaluation, laboratory tests—including plasma and urinary cortisol, ACTH, renin, metanephrines, aldosterone, dexamethasone suppression test and imaging studies (abdominal computed tomography and/or magnetic resonance imaging). Surgical indications included aldosterone or cortisol-secreting adenomas, pheochromocytomas, adrenal cysts or myelolipomas, and adrenocortical tumor.

An enhanced recovery after surgery (ERAS) protocol was applied (185 patients, 64.7%) regardless of the surgical approach used [8]. All procedures were performed under general anesthesia with orotracheal intubation, and patients received prophylactic antibiotics (intravenous cefazolin 2 g) at induction. Postoperatively, patients were administered intravenous fluids and, when indicated, prophylactic antithrombotic therapy (heparin sodium 4000 U.I. subcutaneously) until discharge. In patients with cortisol-secreting tumors, perioperative corticosteroid replacement was provided according to a standardized therapeutic protocol: intravenous hydrocortisone was initiated intraoperatively, gradually tapered in the postoperative period, and subsequently transitioned to oral administration before discharge.

Surgical drains were routinely removed on the first or second postoperative day.

Follow-up consisted of clinical assessment and laboratory testing on postoperative day 2 and at 1-month, followed thereafter by outpatient visits every six months.

### Data extraction

Data was collected using the software program Excel 2024 (ver. 16.89.1) (Microsoft, Redmond, CA, USA). Data were prospectively collected on an electronic anonymised database containing demographic characteristics, indication for surgery, procedural details, and postoperative complications. Final histopathology information was also obtained. All patients provided written consent on the use of their data. The analysis was then retrospectively conducted; since the study fell under the category of research through the use of anonymised data of existing databases, it did not require proportional or full ethics review and approval. The study was conducted according to the Declaration of Helsinki principles and approved by the Department of Surgery Research Committee. After completing the data extraction, a comparison between patients' demographics, diagnosis, final histology, surgical technique, and post-operative course was made.

### Statistical analysis

Statistical analysis was performed using SPSS 28.0.1.1 (IBM Corporation, Armonk, NY, USA). Categorical variables are presented as percentages, and continuous variables are displayed as mean ± standard deviation and median within ranges. Statistical differences were evaluated using the chi-square test for categorical variables. A *p* value <0.05 (two-tailed) was considered statistically significant.

### Surgical technique

For open adrenalectomies, we used a transperitoneal approach through either a midline or a subcostal laparotomy, with the same steps as described for laparoscopy and robotics. For minimally invasive approaches, patients were placed in the lateral decubitus position. The principles of triangulation were preserved: a 12-mm Hasson trocar was inserted at the umbilical level, complemented by three operative ports (or four ports in right-sided lesions to allow the use of a liver retractor). The trocars were positioned radially, extending from the lateral border of the rectus abdominis to the anterior or mid-axillary line.

On the left side, surgical exposure was obtained by mobilizing the splenic flexure of the colon and dividing the splenocolic ligament, followed by medial mobilization of the spleen and pancreatic body and tail. This allowed direct visualization of the superior margin of the left renal vein, where the left adrenal vein was identified, ligated, and divided at its origin. The adrenal gland was then carefully dissected free from surrounding structures and retrieved in an endoscopic specimen bag.

On the right side, the triangular and coronary hepatic ligaments were divided to mobilize the right lobe of the liver and expose the lateral aspect of the inferior vena cava (IVC). This maneuver enabled direct identification, ligation, and division of the right adrenal vein at its origin from the IVC. The adrenal gland was subsequently mobilized and extracted with an endoscopic retrieval bag.

Throughout the procedure, advanced energy devices (Harmonic®, Ethicon) were employed for dissection, and Hem-o-lok® polymer clips were systematically used for secure vascular control.

## Results

A total of 292 adrenalectomies were performed during the study period, with a mean of 18 procedures per year. The cohort included 165 females (56%) and 127 males (44%). A slight sex-related difference in disease incidence was observed without reaching significance (*p* = ns). Mean age at presentation was 54.52 ± 12.6 years (15–89 years). One hundred and seventy one (59%) tumors affected the left side, and 121 (41%) the right side.

Indications for surgery consisted predominantly of functional tumors; 109 (37%) patients were affected with hyperaldosteronism, 84 (29%) patients with Cushing's syndrome, and 43 (15%) with pheochromocytomas. In 50 patients the diagnosis was incidental, and 6 (2%) patients had adrenal metastases having a prior history of malignancy. Specifically, 5 (83%) had adrenal metastases originating from renal cancer, while 1 (17%) had a metastasis from lung cancer. Among non-functioning adrenal lesions, we observed 22 cases of hyperplasia (7.5%), 5 adrenal cysts (1.7%), and 11 myelolipomas (3.8%). Twelve (4%) patients were affected with adrenocortical carcinoma.

Baseline demographic and clinical characteristics of the overall cohort are summarized in Table 1.

**Table 1:** Baseline demographic and clinical characteristics of the overall cohort.

Variable	Overall cohort
Age (years, mean ± SD)	54.5 ± 12.6
Female sex, n (%)	165 (56%)
Male sex, n (%)	127 (44%)
BMI (kg/m^2^, mean ± SD)	27 ± 4
Left-sided tumor, n (%)	171 (59%)
Right-sided tumor, n (%)	121 (41%)
Tumor size (mm, mean ± SD)	46 ± 25

No intra- o postoperative mortality occurred. Laparoscopy represented the most frequently used approach, performed in 223 (76%) patients, followed by open surgery in 30 (10%), robotic adrenalectomy in 27 (9%), the latter progressively increasing, starting from 2020. Overall, in 7 (2%) patients, a conversion from minimally invasive to open approach was required. Furthermore, 6 (50%) patients affected with adrenocortical carcinoma were treated via an open approach, 4 (34%) were managed laparoscopically, 1 (8%) was treated via a robotic approach, and in 1 (8%) case, laparoscopic surgery required conversion to an open adrenalectomy.

When the cohort was stratified into two time periods (2009–2020 and 2021–2025), a clear shift in surgical practice emerged. During the period from 2009 through 2020, laparoscopic adrenalectomy represented the predominant approach, performed in 222 patients (87%), whereas open surgery accounted for 25 cases (10%) and robotic adrenalectomy for 7 (3%).

In contrast, during the 2021–2025 period, robotic adrenalectomy became the most frequently adopted technique, accounting for 24 procedures (63%), followed by laparoscopic surgery in 9 patients (24%) and open surgery in 5 (13%).

Patients who underwent laparoscopic procedures had a mean BMI of 26 ± 2 kg/m^2^, those who underwent open surgery had a mean BMI of 29 ± 4 kg/m^2^, and patients treated with the robotic approach had a mean BMI of 27 ± 4 kg/m^2^ (*p* = ns). Overall operative time was 97 ± 45 min (40–360 min); median 90 min. A statistically significant correlation was observed between the surgical approach and the operative time. The fastest approach was reached using MIS. Specifically, laparoscopy had the shortest mean operative time (82 ± 26 min), significantly lower than robotic surgery (124 ± 26 min; *p* < 0.05). However, both MIS approaches were considerably faster than the open approach, which showed a mean operative time of 175 ± 46 min. (*p* < 0.05). Noticeably, incidentalomas showed a mean diameter of 70 ± 40 mm; (56–190 mm) and the longest mean operative time (147 ± 64 min) when compared with the other adrenal lesions (*p* < 0.05).

Overall, mean blood loss was 91 ± 94 mL (20–700 mL); median 70 mL. Robotic adrenalectomy had the lowest mean intraoperative blood loss (62 ± 20 mL), followed by laparoscopy (66 ± 28 mL), whereas open surgery resulted in markedly higher values (320 ± 179 mL) *p* < 0.05.

Mean size of the tumor was 46 ± 25 mm (10–19 mm); median 40 mm. Two hundreds and eleven (72%) adrenal lesions with a mean diameter of 50 mm or less were always resected via a minimally invasive approach, whereas 9 (3%) measuring 100 mm or more on average were treated through an open procedure. However, we treated with MIS 2 (0.7%) lesions greater than 120 mm.

The most common histological diagnosis was macronodular hyperplasia, identified in 98 patients (34%), followed by adenoma in 87 (30%) and pheochromocytoma in 46 (16%). Malignant disease was found in 23 patients (8%), including 15 adrenocortical carcinomas (5%), and 6 metastases (3%). Interestingly, adenomas were the most frequent diagnosis among females [*n* = 64 (39%) patients], whereas macronodular hyperplasia affected more males than females [*n* = 47 (37%) patients], but no statistical differences were recorded. Intraoperative outcomes by surgical approach are reported in Table 2.

**Table 2:** Intraoperative outcomes by surgical approach.

Variable	Laparoscopic	Open	Robotic	P value
Operative time (min, mean ± SD)	82 ± 26	175 ± 46	124 ± 26	<0.05
Blood loss (mL, mean ± SD)	66 ± 28	320 ± 179	62 ± 20	<0.05
Conversion to open, n (%)	7 (3%)	–	–	NA

The mean length of hospital stay (LOS) was 4 ± 2 days, comparable between the two MIS techniques, but significantly shorter than the open approach, which required an average of 6 ± 3 days (*p* < 0.05).

Minor complications (Clavien–Dindo grade I) occurred in 13 (5%) patients, and resolved without the need for specific treatment. Grade II complications were observed in 12 (4%) patients and consisted of nosocomial or abdominal infections requiring antibiotic therapy; 1 case (0.4%) required interventional radiology drainage of a collection (grade III); all cases were successfully managed.

Postoperative outcomes stratified by surgical approach are reported in Table 3.

**Table 3:** Postoperative outcomes stratified by surgical approach.

Variable	Open	Laparoscopic	Robotic	P value
Length of stay, days (mean ± SD)	6 ± 3	4 ± 2	4 ± 2	<0.05
Clavien–Dindo I–II grades, n (%)	10 (33%)	14 (6%)	1 (4%)	NS
Mortality	0	0	0	NS

## Discussion

Laparoscopic adrenalectomy, first performed by Gagner in 1991, has become the gold standard for the management of most adrenal tumors [9]. Our approach is transperitoneal; however literature shows that even retroperitoneal surgery is associated with very low rates of perioperative complications [10]. Minimally invasive approaches, either laparoscopic or robotic, are associated with shorter operative time, reduced blood loss, and shorter hospital stay compared with open surgery, without compromising safety or efficacy. However, robotic adrenalectomy shows a relative superiority in terms of safety and feasibility, a benefit that is expected to further increase with the improvement of the learning curve and the progressive reduction of costs [11], [12].

Operative time in our cohort was significantly influenced by tumor size and type of surgery. Incidentalomas were associated with the longest operative times and the largest mean diameter, confirming that lesion size remains a key determinant of technical difficulty [13].

Moreover, we observed a significant difference between laparoscopic and robotic approaches, with laparoscopy showing the shortest mean operative time, whereas robotic procedures were slightly longer [14]. These findings reflect current evidence suggesting that robotic adrenalectomy, while more time-consuming, offers advantages in terms of precision and perioperative outcomes, particularly in challenging cases [15], [16]. It should also be noted that the longer operative times recorded in robotic cases may partly reflect the early phase of adoption of this technique in our centre, where robotic adrenalectomy was recently introduced. To date, we have performed 27 procedures and considering that approximately 20 cases are reported as the cut-off to achieve proficiency, our series is still within the expected learning curve [15].

Postoperative morbidity was low, with no major complications or intra- and postoperative deaths, and was more closely related to center expertise rather than the surgical technique [16], [17]. This reinforces the concept that adrenalectomy performed in high-volume centers is a safe procedure. Both MIS approaches showed similar blood loss, significantly lower than open surgery. Although the reduction was more marked in the robotic subgroup, the difference did not reach statistical significance, likely due to the relatively small sample size. Nevertheless, robotic technology offers theoretical advantages enhanced 3D vision, improved ergonomics, and finer dissection — which may translate into better hemostasis and safer tumor handling, as previously suggested [6].

Conversions to open surgery were rare in our series 2%, a rate consistent with previous reports [17]. All conversions occurred in the context of malignant disease or significant intraoperative bleeding. In detail, two conversions were required for local infiltration in pheochromocytoma and adrenocortical carcinoma, two were performed due to bleeding that necessitated splenectomy, and two were required for bleeding alone. These findings confirm that, while minimally invasive adrenalectomy is safe and feasible in most cases, conversion remains necessary in selected scenarios, particularly in the setting of oncologic surgery or haemorrhage. Previous studies have similarly highlighted that malignancy and intraoperative bleeding represent the most frequent causes for conversion, underscoring the importance of careful case selection and prompt intraoperative judgment to ensure patient safety [18].

Interestingly, incidentalomas represented more than half of the tumors approached by laparotomy (51%), mostly due to their larger size and the suspicion of malignancy. This reflects the ongoing concern that MIS for large adrenal masses may increase the risk of capsular rupture, incomplete excision, or spillage of malignant cells, potentially favoring local recurrence.

Another noteworthy finding was that MIS patients had on average a lower age and shorter hospital stay, with a mean reduction of two days compared to the open group. This may partly compensate for the higher costs of robotic surgery, given the reduced theatre occupancy, faster recovery, and lower morbidity. Moreover, patients undergoing open and robotic procedures showed higher BMI, supporting the emerging role of robotics in the management of overweight and obese patients [19].

Right-sided lesions, particularly larger ones, were also more often treated with laparotomy in our series, 19 of the 30 open procedures were right-sided, mainly due to lesion size and oncologic suspicion rather than technical limitations in vein control.

This single-center retrospective study evaluated temporal changes in surgical approaches for adrenalectomy over an extended observational period. When the cohort was stratified into two predefined timeframes (2009–2020 and 2021–2025), an apparent evolution in surgical practice was observed. A deeper analysis, however, demonstrates that this evolution reflects a redistribution within minimally invasive surgery (MIS) rather than a progressive shift from open to minimally invasive adrenalectomy.

Indeed, MIS was already widely adopted during the earlier study period, accounting for approximately 90% of procedures, with laparoscopy representing the predominant technique. In the more recent period, the overall rate of MIS remained comparably high, while robotic adrenalectomy progressively emerged as the preferred minimally invasive approach, largely replacing laparoscopy. Importantly, this transition did not translate into a reduction in open adrenalectomy, whose rate remained relatively stable over time.

The persistence of open surgery suggests that it continues to play a crucial role in selected cases, namely in patients with large tumors, suspected malignancy, or locally advanced disease, where oncological principles and adequate surgical exposure remain essential. Therefore, the temporal changes observed in our series should be interpreted as a technological transition within MIS rather than an expansion of minimally invasive indications.

Several limitations should be acknowledged, including the retrospective design, the single-center nature of the study, and the relatively small number of robotic procedures, which may limit the statistical power of comparative analyses. Nevertheless, the strength of this study lies in the large overall cohort, the long follow-up period, and the consistent surgical expertise and standardized perioperative management typical of a high-volume referral center.

In conclusion, the evolution of adrenalectomy observed in our single-center experience does not reflect a progressive increase in minimally invasive surgery, which was already widely adopted in the early study period, but rather a redistribution within minimally invasive techniques, with robotic adrenalectomy progressively replacing laparoscopy. Importantly, open adrenalectomy maintained a stable role over time, remaining a deliberate and essential option for selected complex tumor cases, including large lesions and suspected malignancy. These findings highlight that the implementation of laparoscopic and robotic adrenalectomy should be guided by careful case selection, institutional expertise, and oncologic principles, rather than by the pursuit of technology adoption alone. For centers wishing to take on robotic adrenalectomy, the key message is that successful program development relies on the balanced integration of laparoscopic, robotic, and open approaches within a patient-centered and safety-driven surgical strategy, tailored to institutional expertise and case complexity.

## CRediT authorship contribution statement

**Gaia Cicioni:** Writing – original draft, Data curation. **Immacolata Iannone:** Writing – original draft, Data curation. **Daniele Crocetti:** Writing – review & editing, Methodology. **Mariarita Tarallo:** Writing – review & editing, Methodology, Data curation. **Paolo Sapienza:** Writing – original draft, Methodology, Investigation, Formal analysis, Data curation, Conceptualization. **Giuseppe Cavallaro:** Writing – review & editing, Data curation. **Giorgio De Toma:** Writing – review & editing, Methodology, Data curation, Conceptualization. **Luigi Petramala:** Writing – review & editing, Methodology. **Claudio Letizia:** Writing – review & editing, Methodology. **Maria Irene Bellini:** Writing – original draft, Methodology, Formal analysis, Conceptualization.

## Ethics approval

Institutional Review Board from Sapienza University, Rome, IT.

## Funding sources

N/A.

## Declaration of competing interest

N/A.
